# Hot Water Cures

**Published:** 1891-11

**Authors:** 


### HOT WATER CURES.

There are but few cases of illness where water should not occupy the highest place as a remedial agent. A strip of flannel or a napkin wrung out of hot water and applied around the neck of a child that has croup, will usually bring relief in ten minutes. A towel, folded several times, quickly wrung out of hot water and applied over the seat of pain in toothache or neuralgia, will generally afford prompt relief. The treatment in colic works like magic. We have known cases that have resisted other treatment for hours yield to this in ten minutes. There is nothing that will so promptly cut short congestion of the lungs, sore throat, or rheumatism as hot water when applied promptly and thoroughly. Pieces of cotton batting dipped in hot water, and kept applied to sores, and new cuts, bruises, and sprains, is the treatment adopted in many hospitals. Sprained ankles have been cured in an hour by showering them with hot water, poured from a height of a few feet. Tepid water acts promptly as an emetic, and hot water taken freely half an hour before bedtime is the best cathartic in case of constipation.—*Leeds Mercury*.

